# Positive Prospective Mental Imagery Characteristics in Young Adults and Their Associations with Depressive Symptoms

**DOI:** 10.1007/s10608-023-10378-5

**Published:** 2023-04-28

**Authors:** Marta Anna Marciniak, Lilly Shanahan, Harald Binder, Raffael Kalisch, Birgit Kleim

**Affiliations:** 1 Department of Psychology, University of Zurich, Lenggstrasse 31, Zurich, 8032 Switzerland; 2 Department of Psychiatry, Psychotherapy and Psychosomatics, Psychiatric University Hospital (PUK), University of Zurich, Zurich, Switzerland; 3 Jacobs Center for Productive Youth Development, University of Zurich, Zurich, Switzerland; 4 Institute of Medical Biometry and Statistics, Faculty of Medicine and Medical Center, University of Freiburg, Freiburg, Germany; 5 Freiburg Center for Data Analysis and Modelling, University of Freiburg, Freiburg, Germany; 6 Leibniz Institute for Resilience Research (LIR), Mainz, Germany; 7 Neuroimaging Center (NIC), Focus Program Translational Neuroscience (FTN), Johannes Gutenberg University Medical Center, Mainz, Germany

**Keywords:** Mental imagery, Positive prospective mental imagery, Vividness, Ease, Mobile app, Depression

## Abstract

**Background:**

Positive prospective mental imagery plays an important role in mental well-being, and depressive symptoms have been associated with difficulties in generating positive prospective mental images (PPMIs). We used a mobile app to gather PPMIs generated by young adults during the COVID-19 pandemic and analyzed content, characteristics, and associations with depressive symptoms.

**Methods:**

This is a secondary analysis of a randomized controlled trial with 95 healthy young adults allocated into two groups (intervention and control). Participants used the mobile app decreasing mental health symptoms for seven consecutive days. Fifty participants in the intervention group reported PPMIs at least three times per day using a mobile app inducing PPMI generation. We categorized entries into themes and applied moderation models to investigate associations between PPMI characteristics and depressive symptoms.

**Results:**

We distinguished 25 PPMI themes. The most frequent were related to consuming food and drinks, watching TV/streaming platforms, and doing sports. Vividness and ease of generation of PPMIs, but not their anticipation, pleasure intensity or number of engagements with the app were associated with fewer depressive symptoms.

**Conclusions:**

We identified PPMI themes in young adults and found significant negative associations between depressive symptoms and vividness and generation ease of PPMIs. These results may inform prevention and intervention science, including the design of personalized interventions. We discuss implications for future studies and treatment development for individuals experiencing diminished PPMI.

**Supplementary Information:**

The online version contains supplementary material available at 10.1007/s10608-023-10378-5.

## Introduction

Positive prospective mental imagery refers to mental simulation of positive future events (Holmes et al., 2009, 2016; Ji et al., 2019) and thus relates to the ability to project oneself forward in time to pre-experience a pleasant event (Atance & O’Neill, 2001). An impoverished capacity for, or the inability to generate PPMIs is associated with depression (Holmes et al., 2016; Roepke & Seligman, 2016). Individuals with depressive symptoms often generate fewer positive prospective mental images (PPMIs) than those with fewer or without depressive symptoms, and participants with sub-clinical depressive symptoms have demonstrated reduced implicit (i.e., automatic, without conscious control) positive future expectancies compared to healthy controls (Kosnes et al., 2013; MacLeod et al., 1997; MacLeod & Byrne, 1996). An elevated level of depressive symptoms has also been associated with the generation of more negative and fewer positive future events and impairments in spontaneous generation of PPMI (Holmes et al., 2016). Individuals with depressive symptoms mentally simulate PPMI with other characteristics, namely lower specificity, less detail and vividness and less use of the first-person perspective when describing their images than those without depression (Hallford et al., 2020; Morina et al., 2011; Szőllősi et al., 2015). Individuals with depression also generate PPMIs at lower speed and less detailed compared to non-depressed controls (Stöber, 2000). This is also true of dysphoric individuals, who show poorer vividness of positive imagined future events (Anderson & Evans, 2015).

Mental imagery may be a key driver for successful mental health interventions, such as imagery rescripting or imaginal exposure therapy (Pile et al., 2021). It plays an important role in treating depression and anxiety (Holmes et al., 2009, 2016; Torkan et al., 2014), specifically by decreasing the number and vividness of negative images and increasing the number of positive images. This, in turn, boosts behavioral activation, as imagining one’s own future positive behavior increases the likelihood of that behavior being enacted (Chan & Cameron, 2012; Renner et al., 2017). Indeed, the recent reports confirm that PPMI can boost reward sensitivity (Ji et al., 2021; Linke & Wessa, 2017), what leads to increased engagement in reward-motivated activities and, consequently, fewer depressive symptoms (Renner et al., 2021). This is especially important for people who experience large amounts of stress, which can lead to lower reward sensitivity and, in turn, anhedonia, one of the main symptoms of depression (Bogdan & Pizzagalli, 2006). In terms of potential mechanisms, PPMI has been shown to stabilize mood in bipolar disorder (Holmes et al., 2011), decrease dysphoria symptoms (Boland et al., 2018; Grol et al., 2017; Holmes et al., 2008; Pictet et al., 2011), reduce anxiety about public speaking (Landkroon et al., 2022), and increase optimism (Ji et al., 2017) by modifying cognitive biases and creating more positive and benign interpretations of negative life events, what enhances the positive appraisal style and stress resilience (Kalisch et al., 2015). Importantly, the capacity to generate PPMIs may also benefit healthy populations, as PPMIs have been found to be more effective than verbal thoughts in evoking positive emotional responses (Holmes & Mathews, 2010) and to reduce worry (Skodzik et al., 2017) and stress (Watanabe et al., 2006). Mental imagery is helpful in reducing procrastination and increasing empathy (Blouin-Hudon & Pychyl, 2017).

During the COVID-19 pandemic, the recall and generation of mental images of realistic positive future experiences might have been particularly challenging for many, as access to and availability of everyday pleasurable events and situations were often greatly limited (Diaz Hernandez et al., 2021) and related to many closed facilities, including cultural institutions, shops, and sports facilities, as well as highly limited social contacts. This, in turn, reduced the frequency of occurrence of positive events in daily life and consequently, reduced the opportunity for imaging of prospective positive experiences. Such an inability to imagine positive experiences may have contributed to the rise in psychiatric symptoms and diagnoses during the pandemic (Gobbi et al., 2020) and to negative impacts of new and unique daily stressors on vulnerable individuals (Veer et al., 2021). The long-term effects of these circumstances on mental health remain unknown (Bourmistrova et al., 2022; Sampogna et al., 2022). Positive prospective mental imagery and the capacity to imagine pleasurable events could be used in preventive or therapeutic settings to reduce the negative impact of stressors on psychological well-being.

For this study, we investigated PPMIs reported by the intervention group of a randomized controlled trial (RCT) which aimed to assess the efficacy of an ecological momentary intervention (EMI) – Imager. The full RCT included 95 participants (50 in the intervention group and 45 in the control group). Participants in the intervention group practiced and reported PPMIs with the use of an EMI app, while the control group has not received mental imagery training, and only reported their affective state with the use of another, same-looking mobile app (hence, the control group is not included in the current report). The results of RCT suggested that Imager was efficacious in decreasing depressive symptoms (medium effect size for a time x group interaction, very large main effect of time for the intervention group, and small main effect of time for the control group), and perceived level of stress (small-to-medium effect size for a time x group interaction, large effect size for main effect of time in the intervention group, and small effect of time in the control group). A full description of the study and results can be found in (Marciniak et al., 2022).

In the current, secondary analysis, we took on a qualitative approach and conducted a thematic analysis of reported PPMIs and investigated PPMI-specific characteristics (vividness, pleasure intensity, anticipation, ease of generation) in order to better understand the factors contributing to the decrease of depressive symptoms in mental imagery intervention.

As the first step, we conducted a thematic analysis and a frequency analysis of PPMIs. Subsequently, we estimated the average rates of vividness, pleasure intensity, anticipation as well as ease of generation for each of the PPMI themes. Based on previous reports, e.g., (Anderson & Evans, 2015; Hallford et al., 2020; Szőllősi et al., 2015), we further hypothesized that these mental imagery characteristics (vividness, pleasure intensity, anticipation, ease of generation):

- would be positively correlated with each other, and,
- would moderate a reduction in depressive symptoms after one week of EMI use.

In an exploratory manner, we tested:

c) whether the number of engagements with the app (i.e., the number of all completed PPMI surveys by each participant) would moderate a decrease in depressive symptoms, and,
d) whether there would be any association between the most reported theme by the participant and the diminishment of the depressive symptoms.

A better understanding of the type and characteristics of PPMIs experienced by individuals and of their relationships to depressive symptoms can inform innovative interventions for vulnerable individuals or those with difficulties generating PPMIs and optimize current prevention and intervention strategies beyond the pandemic.

## Methods

### Materials

#### mHealth Ecological Momentary Intervention – Imager

**Fig. 1:**
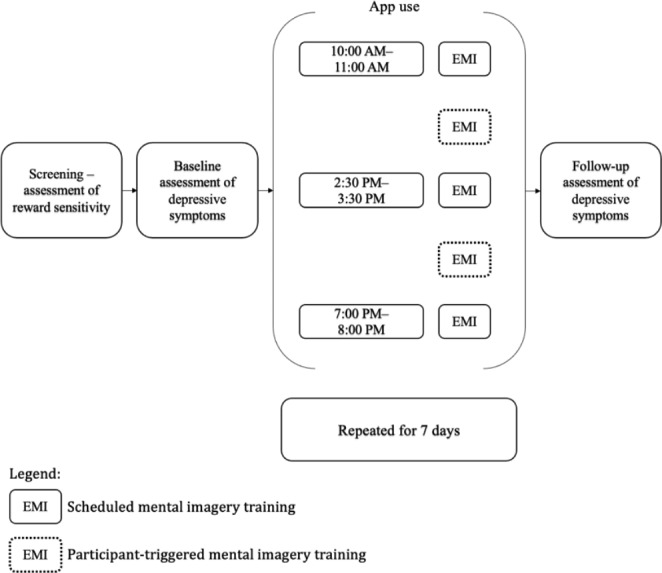
Study Procedure

Imager is a mobile app developed to increase mental imagery skills. Imager sent ten prompts per day, between 8:30 AM and 11:00 PM to assess how the participants felt at the moment. Participants were presented with 13 items assessing their affect (e.g., I feel cheerful, I feel stressed, I can concentrate well) rated on a scale from 1 (not at all) to 7 (very much), and questions on their social and physical activity, as well as on the substance use. A preprint that provides a full description of the development of the app, including these measures is available (Marciniak et al., 2022). Out of the ten surveys, three which were sent within one-hour windows at 10:00 AM, 2:30 PM, and 7:00 PM were combined with mental imagery training. Participants were asked to think about something pleasant that could happen to them within the next few hours. Participants were then instructed to create a mental image of the event involving all their senses, including what they would see, hear, taste, touch and smell while being actively involved in this event. At the end of each EMI, participants were asked to provide a short description of their experience of creating a mental image and to evaluate their mental imagery experience. To evaluate vividness, they were asked “How vivid was the image you created?” and answered the question on a Likert scale from 1 (weak) to 7 (almost as vivid as real). Pleasure, “This event will be pleasant for me”, anticipation “I can’t wait for this event to happen” and ease of generation of the image “It was easy for me to imagine the event” were rated on a Likert scale from 1 (strongly disagree) to 7 (strongly agree). Each time, participants had 20 min to complete the EMI. Three EMIs per day appeared by default, and, in addition, participants could trigger an EMI whenever they felt the need for one, with no limit on the number of self-triggered mental imagery training (see Fig. 1).

#### Questionnaires

Depressive symptoms were indexed using the Beck Depression Inventory II (BDI-II) (Beck et al., 1996, German translation by Kühner et al., 2007). This widely used self-report questionnaire consists of 21 items measuring the severity of depression symptomatology (e.g., sleep quality, appetite, feelings of guilt, suicidal thoughts, anhedonia), score range is from 0 to 63, consistency α ≥ 0.84, and retest reliability *r* ≥ .75 after two weeks in nonclinical samples for German translation (Kühner et al., 2007). Baseline level of reward sensitivity was indexed with the German translations of the Reward Responsiveness subscale of the Behavioral Avoidance/Inhibition Scales (BIS/BAS) (includes 4 questions, score range = 4–16, consistency α ≥ 0.72 for the full BAS scale, and retest reliability *r* ≥ .68 for two weeks in nonclinical samples) (Jorm et al., 1998; Strobel et al., 2001). We calculated the reliability in our sample as suggested by (Koo & Li, 2016) with the use of Intraclass Correlation Coefficient (ICC). For BDI-II the reliability was moderate (ICC = 0.59, *p* < .001), and for the Reward Responsiveness scale of BIS/BAS it was good (ICC = 0.78, *p* < .001).

### Procedure

#### Pandemic Context

We enrolled participants between October 2020 and April 2021. This half-year period covered the whole second wave and the beginning of the third wave of the COVID-19 pandemic in Switzerland. At this time, universities were operating remotely. Restaurants, bars, and sports facilities were closed from December 22, 2020 until the end of data collection, and museums were closed from December 22, 2020 and reopened on March 1, 2021. All these venues were either closed or opened on a limited basis in the preceding period. Non-essential shops were open with certain limitations until January 18 and then closed until March 1, 2021.

#### Inclusion Criteria and Recruitment

The Ethics Committee of the Faculty of Arts and Social Sciences of the University of Zurich approved the randomized controlled trial study (#20.6.11), although the focus of this paper lies in the intervention group only.

Participants were recruited from universities based in the greater Zurich area using online methods: announcements on university websites, mailing lists dedicated to students of particular departments, and social media. Inclusion criteria were being (1) fluent in German, (2) a student of higher education institution, (3) between 18 and 29 years old, and (4) scoring 5 points or more on a reversed BIS/BAS Reward Responsiveness scale (Jorm et al., 1998). We wanted to include only participants with lower levels of reward responsiveness, as they could benefit most from participation in the study. The threshold was set after the meeting with stakeholders mirroring the target group. We excluded volunteers with mental illness and those receiving treatment from a qualified psychologist or psychiatrist, as reported by them in the screening form. All participants provided informed consent.

#### Study Timeline

All potential participants interested in the study, filled in a short screening form (online), including basic demographic information and BAS Reward Responsiveness scale. Those who scored 5 points or more on a reversed scale, were included in the study. At the beginning of the study, participants attended a baseline meeting (conducted as an online video call), during which they completed a baseline questionnaire assessment, including BDI-II, and received instructions on how to download and use Imager. They were provided with interactive training in PPMI. During the training, participants were familiarized with the definition and origin of PPMI and instructed on how to implement it in daily life while assisted by the EMI. For instance, participants were asked to prepare a list of possible and realistic pleasant events that may happen to them within the next few days. With the assistance of a trained research assistant who actively oversaw the training, they completed a few PPMI examples (e.g., creating a mental image for a pre-defined scenario) based on the work of Blackwell et al. (2015), Holmes et al. (2016), and Renner et al. (2017), focusing on pleasant events in the near future. Subsequently, participants used Imager for seven consecutive days and received notifications to complete the EMI at least three times per day. After seven days of app use, participants were invited to participate in another online video call (see Fig. 1). In order to ensure data reliability, this call has always taken place on the first day following the EMI use. Participants filled in the BDI-II questionnaire, received debriefing, financial reimbursement, and interactive charts depicting their mood fluctuations during the week of app usage.

### Analysis

For the thematic analysis, as there is no similar literature on the topic, we employed data-driven approach meaning that number of the themes and themes themselves were based on participants’ entries. We coded the EMI entries provided by participants following the Braun and Clarke guidelines for thematic analysis (Braun & Clarke, 2006) without pre-specified themes (Ibrahim, 2012). A qualified psychologist performed three stages of data reduction: (1) organizing data and generating an initial list of themes via a data-driven approach, (2) reviewing the themes, and (3) finalizing the theme set (Miles & Huberman, 1994). An independent psychologist categorized a random set of 20% of entries. Afterwards, the categorizations from two psychologists were compared. The inter-rater reliability was at 97.2%. The raters discussed discrepancies in their ratings and agreed on final themes.

In addition, we tested how PPMI characteristic (vividness, pleasure, anticipation, ease) and total number of engagements with the app moderated the decrease of depressive symptom. Because the BDI-II scores were not normally distributed, we log transformed the data. We used the *nlme* package (Pinheiro et al., 2021) in R (version 4.0.4) for the main within-subject analysis. Time (baseline vs. follow-up) was the main effect in the model. PPMI characteristics with at least three surveys per day nested within a given day, and seven days nested within a person were the moderators, and depressive symptom severity was the dependent variable.

## Results

### Sample

On average, each participant engaged in 28.2 PPMIs in one week. In total, we collected 1,412 PPMIs from 50 participants. The majority of participants were female (80%). The mean age of participants was 21.00 years (*SD* = 1.95), and 94% of the participants were studying psychology (see Table 1).

### Prospective Mental Imagery Themes and Characteristics

We identified 25 PPMI themes. Consuming food and drink (23%), watching TV or streaming platforms (11%), and doing sports (7%) were the most frequently reported (see Table 2).

**Table 1:** *Demographic information*

Demographics	Values
Age - mean (*SD*) [yrs]	21.00 (*SD* = 1.95)
Age - range [yrs]	18–26
% Female	80% (40/50)
% Students	100%
Psychology	94% (47/50)
Other departments	6% (3/50)
Beck Depression Inventory-II - Mean Baseline score (*SD*)	9.72 (*SD* = 6.25)
Beck Depression Inventory-II - Mean Follow-up score (*SD*)	6.76 (*SD* = 5.58)
Number of participants with minimal depression in baseline (0–13 points in BDI-II)	39^a^
Number of participants with mild depression in baseline (14–18 points in BDI-II)	7
Number of participants with moderate depression in baseline (19–29 points in BDI-II)	4
Number of participants with severe depression in baseline (30–63 points in BDI-II)	0
Number of participants with minimal depression in follow-up (0–13 points in BDI-II)	44^b^
Number of participants with mild depression in follow-up (14–18 points in BDI-II)	4
Number of participants with moderate depression in follow-up (19–29 points in BDI-II)	2
Number of participants with severe depression in follow-up (30–63 points in BDI-II)	0

*Note.*^a^39 participants, including 18 participants with scores 2–6, 13 participants with scores 7–10, 8 participants with subthreshold scores 11–13, ^b^44 participants, including 29 participants with scores 0–6, 12 participants with scores 7–10, 3 participants with subthreshold scores 11–13

**Table 2:** *PPMI categories and frequencies*

Theme	Example item	Counts	Proportion
Consuming food/drink	“I will eat my favorite pizza.” “I will drink warm tea.”	326	23%
Watching TV/streaming platforms	“I will watch an Arsenal match.” “I will watch my favorite series, ‘Modern Family’.”	150	11%
Doing Sports	“I will go jogging.”	105	7%
Meeting others	“I will meet my father.”	88	6%
Walking	“I will go for a walk.”	83	6%
Cooking	“I will try a new recipe for Pad Thai.” “I will bake a delicious chocolate cake.”	79	6%
Sleeping/napping	“I will take a 20-minute power nap.” “I will go to sleep after this long day.”	68	5%
Personal hygiene	“I will take a bath.” “I will take a long, hot shower.”	56	4%
Listening to music or audiobooks	“I will listen to my favorite rock music.”	53	4%
Arts and cultural performances	“I’ll will go to choir classes today.” “During lunchtime, I will go to a museum.”	52	4%
Regular duties and chores	“I will listen to a lecture by my favorite professor.”	51	4%
Reading	“I will read ‘Little Women’ in a moment.”	48	3%
Other	“I will drive my car.”	42	3%
Self-care, personal comfort	“I will light candles to make myself feel cozy.”	37	3%
Shopping	“I’ll go buy new body balm in my favorite cosmetics shop.”	31	2%
Playing games	“I am going to play video games with my cousin.”	29	2%
Enjoying nature (not including walking)	“I will take a deep breath of fresh air.” “I will watch snow falling.”	23	2%
Playing with pets	“I will play with my dog.” “I will brush my cat.”	21	1%
Physical contact	“I will fall asleep cuddled by my partner.” “I will kiss my girlfriend.”	19	1%
Passive relaxing	“I will lie on a couch and do nothing.”	18	1%
Phone/video calls	“I will call my mom on Zoom.” “I am going to call my boyfriend and ask how his day was.”	12	1%
Finishing tasks	“I will cross things off my to-do list, because I did them great!”	9	1%
Volunteer work	“I will teach math to a refugee.”	6	0%
Planning/daydreaming	“I will come back home and plan a weekend with my little sister.” “I will dream that my partner is healthy again and I can see him.”	4	0%
Texting	“I will send a cute message to my boyfriend.” “I will write to my friend telling her that I miss her.”	2	0%
Total		1412	100%

Average vividness for all PPMIs was high (*M* = 5.30, *SD* = 1.16). The most vivid PPMIs were related to volunteer work (*M* = 6.00, *SD* = 0.63), playing with pets (*M* = 5.75, *SD* = 0.97), and arts and cultural performances (*M* = 5.71, *SD* = 1.23), while passive relaxing (*M* = 4.46, *SD* = 0.78), finishing tasks (*M* = 4.67, *SD* = 1.12), and regular duties and chores (*M* = 4.68, *SD* = 1.51) were rated the least vivid (see Table 3). Mean PPMI pleasure intensity was also high (*M* = 5.71, *SD* = 1.05). The most pleasant PPMIs were related to physical contact with someone else (*M* = 6.32, *SD* = 0.95), sleeping (*M* = 6.12, *SD* = 1.10), and finishing tasks (*M* = 6.11, *SD* = 0.78). The least pleasant were PPMIs related to regular duties and chores (*M* = 4.49, *SD* = 1.43). All other PPMIs were rated 5 or higher on the scale, including texting (*M* = 5.00, *SD* = 1.41) and shopping (*M* = 5.26, *SD* = 1.18). Average anticipation was *M* = 5.20, *SD* = 1.34. The most anticipated events were volunteer work (*M* = 6.50, *SD* = 0.84), followed by finishing tasks (*M* = 6.33, *SD* = 1.00) and physical contact with others (*M* = 6.05, *SD* = 1.13). The least anticipated PPMIs were regular duties and chores (*M* = 4.04, *SD* = 1.37) and passive relaxing (*M* = 4.17, *SD* = 1.34), followed by shopping (*M* = 4.42, *SD* = 1.57). Average ease of generation of the PPMI was high (*M* = 5.38, *SD* = 1.22). The easiest events to imagine were related to arts and cultural performances (*M* = 5.92, *SD* = 0.1.04), playing with pets (*M* = 5.75, *SD* = 0.91) and going for a walk (*M* = 5.63, *SD* = 0.97). Passive relaxing (*M* = 4.89, *SD* = 1.08), playing games (*M* = 5.00, *SD* = 1.63) and phone/video calls (*M* = 5.09, *SD* = 1.45; see Table 3) were somewhat less easy to imagine.

**Table 3:** *Vividness, pleasure, anticipation, and ease scores for each PPMI theme*

Theme	Vividness		Pleasure		Anticipation		Ease	
	Mean	*SD*	Mean	*SD*	Mean	*SD*	Mean	*SD*
Consuming food/drink	5.27	1.10	5.94	0.89	5.47	1.20	5.38	1.23
Watching TV/streaming platforms	5.22	1.27	5.65	1.14	5.16	1.45	5.23	1.35
Doing Sports	5.31	1.08	5.32	1.10	4.84	1.30	5.38	1.23
Meeting others	5.48	1.20	5.91	1.07	5.60	1.17	5.44	1.11
Walking	5.54	1.10	5.73	0.96	4.91	1.29	5.63	0.97
Cooking	5.42	0.98	5.57	0.84	5.10	1.18	5.41	1.06
Sleeping/napping	5.18	1.26	6.12	1.10	5.90	1.27	5.38	1.40
Personal hygiene	5.45	0.78	5.70	0.89	4.70	1.35	5.52	0.87
Listening to music or audiobooks	5.19	1.13	5.68	0.80	5.06	1.10	5.21	1.32
Arts and cultural performances	5.71	1.23	5.92	1.03	5.42	1.38	5.92	1.04
Regular duties and chores	4.78	1.28	4.49	1.43	4.04	1.37	5.29	1.24
Reading	5.09	1.08	5.66	0.76	4.91	1.06	5.51	1.04
Other	5.31	1.00	5.38	1.10	5.07	1.42	5.21	1.14
Self-care, personal comfort	5.57	1.40	5.46	1.16	4.95	1.58	5.16	1.37
Shopping	4.68	1.51	5.26	1.18	4.42	1.57	5.16	1.55
Playing games	5.21	1.15	5.79	1.11	5.41	1.32	5.00	1.63
Enjoying nature (not including walking)	5.52	0.95	5.78	0.80	5.30	1.15	5.57	1.20
Playing with pets	5.75	0.97	6.00	0.92	5.60	1.35	5.75	0.91
Physical contact	5.58	1.74	6.32	0.95	6.05	1.13	5.37	1.38
Passive relaxing	4.61	0.78	6.06	0.80	4.17	1.34	4.89	1.08
Phone/video calls	5.45	1.29	6.09	0.70	5.36	1.12	5.09	1.45
Finishing tasks	4.67	1.12	6.11	0.78	6.33	1.00	5.11	0.93
Volunteer work	6.00	0.63	5.83	0.75	6.50	0.84	5.50	0.84
Planning/daydreaming	5.25	1.71	5.75	0.96	5.25	1.50	5.25	1.71
Texting	5.50	0.71	5.00	1.41	5.50	0.71	5.50	0.71

Table 4 shows the correlations between PPMI characteristics. The highest correlations were between vividness and ease of generation (*r* = .86, *p* < .001) and between pleasure intensity and anticipation (*r* = .74, *p* < .001).

**Table 4:** *Correlations between positive prospective mental imagery characteristics*

	Vividness	Pleasure	Anticipation
Pleasure	0.46 ***		
Anticipation	0.55 ***	0.74 ***	
Ease	0.86 ***	0.52 ***	0.62 ***

*Note.* *** *p* < .001

### Associations Between PPMI Characteristics and Depressive Symptoms

The results from the RCT where the effects of the use of Imager on depressive symptoms were tested against an active control group are available elsewhere (Marciniak et al., 2022). Overall, Imager has proved to be effective in decreasing depressive symptoms as compared to an active control group (Group x Time interaction was significant, β = −0.34, *df* = 93, *p* = .004, Cohen’s *f* = 0.27, 90% CI [0.10, 0.40] / η_p_^2^ = 0.09, 90% CI [0.02, 0.19]).

Here we regressed the change in depressive symptoms over the week using Imager on PPMI characteristics. Intra-class correlation coefficient of ICC(1) = 0.57, *p* < .001, ICC(2) = 0.72 for the participant level of BDI-II outcomes, confirmed the hierarchical structure of the data and justified the use of linear mixed models for moderation analysis.

PPMI vividness and ease of generation were significant moderators for the decrease in depressive symptoms over the course of the week: (β = − 0.28, *df* = 48, *p* = .024, Cohen’s *f* = 0.34; 90% CI [0.09, 0.58] for vividness, and β = −0.32, *df* = 48, *p* = .005, Cohen’s *f* = 0.43; 90% CI [0.18, 0.67] for ease of generation.) Decrease in depressive symptoms was not moderated by pleasure (β = − 0.08, *df* = 48, *p* = .645, Cohen’s *f* = 0.07; 90% CI [0.00, 0.30]), anticipation (β = − 0.16, *df* = 48, *p* = .167, Cohen’s *f* = 0.20; 90% CI [0.00, 0.44]), or number of engagements with the app (β = − 0.02, *df* = 48, *p* = .106, Cohen’s *f* = 0.24; 90% CI [0.00, 0.48]) (see Table 5).

**Table 5:** *Statistical models*

Model type	Variable of interest	Value (β)	*SE*	*DF*	*t* value	*p* value	Cohen’s *f* [90% CI]
Moderation	**Vividness**						
	Intercept	2.21	0.08	49	26.91	< 0.001	
	Time	1.06	0.65	48	1.64	0.109	0.84 [0.56, 1.11]
	Vividness	-0.28	0.12	48	-2.33	0.024	0.34 [0.09, 0.58]
Moderation	**Ease**						
	Intercept	2.21	0.08	49	26.91	< 0.001	
	Time	1.28	0.59	48	2.17	0.035	0.86 [0.58, 1.13]
	Ease	-0.32	0.11	48	-2.95	0.005	0.43 [0.18, 0.67]
Moderation	**Anticipation**						
	Intercept	2.21	0.08	49	26.91	< 0.001	
	Time	0.37	0.58	48	0.63	0.533	0.82 [0.54, 1.09]
	Anticipation	-0.16	0.11	48	-1.40	0.167	0.20 [0.00, 0.44]
Moderation	**Pleasure**						
	Intercept	2.21	0.08	49	26.91	< 0.001	
	Time	0.01	0.96	48	0.01	1.000	0.80 [0.53, 1.07]
	Pleasure	-0.08	0.17	48	-0.46	0.645	0.07 [0.00, 0.30]
Moderation	**Engagement**						
	Intercept	2.21	0.08	49	26.91	< 0.001	
	Time	0.02	0.29	48	0.07	0.942	0.82 [0.54, 1.09]
	Engagement	-0.02	0.01	48	-1.65	0.106	0.24 [0.00, 0.48]

In addition, we investigated whether there was any association between the most reported themes by the participants and the decrease in depressive symptoms. For 28 participants, the theme of consuming food/drink was the most reported theme, while for other participants the most reported themes were: watching TV/streaming platforms (*N* = for 8 participants), self-care (*N* = 3), arts and cultural performances (*N* = 2), walking (*N* = 2), volunteer work (*N* = 1), phone/video calls (*N* = 1), finishing duties (*N* = 1), sleeping (*N* = 1), cooking (*N* = 1), passive relaxing (*N* = 1), and other (*N* = 1) (see Table 6). The most notable decrease in depressive symptoms (and with the potential clinical significance) was observed in participants whose most reported PPMI themes were related to arts and cultural performances (decrease of 12.50 points in follow-up versus baseline) and walking (decrease of 6.50 points in follow-up versus baseline). However, both these themes were the most reported by two participants each, hence the outcomes must be interpreted with caution and should be treated as preliminary.

**Table 6:** *Changes in depressive symptoms per most reported theme*

Most reported theme	Beck Depression Inventory-II - *Mean Baseline score (SD)*	Beck Depression Inventory-II - *Mean Follow-up score (SD)*	Change in raw score within participants
Consuming food/drink	9.64 (*SD* = 5.22)	6.79 (*SD* = 4.80)	-2.85
Watching TV/streaming platforms	11.38 (*SD* = 7.82)	8.38 (*SD* = 6.78)	-3
Self-care	11.33 (*SD* = 5.13)	11.67 (*SD* = 10.69)	+ 0.34
Arts and cultural performances	23 (*SD* = 7.07)	10.50 (*SD* = 7.78)	-12.50
Walking	8 (*SD* = 4.24)	1.5 (*SD* = 0.71)	-6.50
Volunteer work	7 (1 participant)	5 (1 participant)	-2
Phone/video calls	2 (1 participant)	0 (1 participant)	-2
Finishing duties	5 (1 participant)	2 (1 participant)	-3
Sleeping	5 (1 participant)	4 (1 participant)	-1
Cooking	2 (1 participant)	2 (1 participant)	0
Passive relaxing	6 (1 participant)	6 (1 participant)	0
Other	3 (1 participant)	3 (1 participant)	0

## Discussion

### Summary and Integration of Findings

We collected mental images of positive future events reported by young adults across one week during the COVID-19 pandemic using an ecological momentary approach and investigated the associations between PPMI characteristics and depressive symptoms. Most reported PPMIs were related to consuming food and drinks, watching TV or streaming platforms, and doing sports. The highest diminishment of depressive symptoms was observed in participants who mostly reported PPMIs related to arts and cultural performances as well as walking, however, those are preliminary results that should be interpreted with caution and replicated in further studies.

Some PPMIs were experienced as more vivid and easier to generate, such as volunteer work, playing with pets, arts and cultural performances, physical contact with others, and enjoying nature. However, all PPMIs were experienced as rather vivid, pleasant, anticipated, and easy to generate, as documented by ratings of four points or higher on a 7-point scale. This was expected for pleasure and anticipation, as participants were instructed to create positive PPMI, but not for vividness and ease of generation. Most reported PPMIs were related to participants being alone. This may be related to the COVID-19 pandemic leading through social measures to limited social interactions and is not associated with measurement artifacts, as participants indicated being alone in around half of EMI surveys (51.5%). The most vivid and easiest to generate PPMIs, however, mostly involved interactions with people or animals. Such PPMIs were generated less frequently compared to some of the other categories, again, possibly due to social distancing and other regulations during the pandemic. This confirms previous findings (e.g., Karatzias et al., 2009; Morina et al., 2013; Wilson et al., 2018) that vivid positive mental imagery may help reduce psychopathology symptoms, including depressive symptoms.

Generation ease is a relatively new term in mental imagery research and is mostly used to assess cognitive tasks and paradigms (Lanata et al., 2020; Nagels-Coune et al., 2020). Previous studies suggest that availability of mental images is dependent on their concreteness (e.g., Paivio 1969), but the ease of generation of PPMI as a separate construct has not yet been researched in connection with psychopathology symptoms. Some researchers do not separately index generation ease and vividness (Tiba, 2018). While our study found significant correlation between vividness and generation ease, there were also important differences. As indicated by the effect size, generation ease showed greater association with reduction of depressive symptoms over the course of the week than vividness. Our analysis of the PPMI characteristics could facilitate new paths for investigating not only the vividness of PPMIs but also the ease of image generation and the relation of these characteristics to both depressive symptomatology and each other.

### Limitations

Our study is not free from limitations. First, we did not collect the data outside the COVID-19 pandemic setting, which could have had an impact on the type of PPMIs reported by participants. As mentioned above, the pandemic regulations significantly impacted participants’ life and reduced engagement in desirable and pleasant activities, and social contacts, therefore limiting the scope of PPMIs presented in the current manuscript, as such events were less likely to realize. We recommend a replication of the study procedure outside the pandemic setting to investigate the similarities and differences between reported PPMIs. Second, most participants were healthy female psychology students with relatively low levels of depressive symptoms (i.e., below clinical cut-off scores), so the results cannot be generalized to either general or clinical populations. Future studies should focus on more diverse populations in terms of gender, age, and psychopathology. We also recommend gathering reports of specific PPMIs experienced at times of elevated depressive symptoms to further explore the role of ease of generation and vividness in reducing such symptoms in clinical samples. We did not gather data on whether the events imagined during the mental imagery training have taken place later the same day and whether they were similar to the images created by participants, what limits possible conclusions on emotional impact of mental imagery on the actual real-life experiences. Moreover, by reporting PPMIs a few times per day for a whole week, participants may have improved their mental imagery skills and reduced depressive symptoms, as indicated in previous reports on the role of PPMI and mental imagery in psychopathology (e.g., Ji et al., 2017; Linke & Wessa, 2017; Renner et al., 2017), however, our results suggest that there was no connection between number of engagements with the app (i.e., the number of all completed PPMI surveys by each participant) and change in depressive symptoms, and the only significant moderators were qualitative in nature (especially vividness which directly translates to the quality of the image). More studies, preferably using real-life data sampling rather than laboratory-induced mental imagery in clinical samples, are needed to clarify these associations.

### Strengths

Despite these limitations, our study has several strengths. We used a smartphone app that allowed us to gather PPMIs repeatedly during the day for one week in the natural environment of participants and, arguably, without disrupting their daily routines. This type of data sampling also minimizes recall bias, as participants were prompted a few times throughout the day and could use the app at their own pace, which helped them report PPMIs immediately after they occurred. Such validity would not have been possible to achieve via laboratory procedures. To the best of our knowledge, our study is the first to investigate themes of PPMI with ecological momentary data collected via an app and to consider ease of generating PPMIs as associated to the reduction of the depressive symptoms.

### Practical Implications

The analysis presented in this paper could have important practical implications and may inform preventive mental health workshops, the design of personalized standalone interventions for individuals with PPMI deficits, or add-on training to therapy sessions. The thematic summary of PPMIs could constitute a basis for mental imagery training by facilitating the generation of PPMIs by individuals experiencing difficulties in spontaneous generation of mental images. Such actions could be especially relevant for young adults, who constituted the sample for this study. PPMI may initiate behavioral activation, a crucial component of cognitive behavioral therapy and an important factor in depression prevention and treatment. Studies have suggested that individuals who imagine themselves engaging in certain activities are more likely to pursue those activities in the future (Chan & Cameron, 2012; Renner et al., 2017). The most frequent PPMIs were, however, related to sedentary behavior (eating, drinking, watching TV, and streaming platforms), which, if overused, could hamper individual’s health and mental well-being. After replications of the study procedure on both healthy and clinical samples, which would allow us to better understand the results and mechanisms underlying the diminishment of depressive symptoms in relation to PPMI characteristics and themes, the summary of the PPMIs could increase the benefits of mental imagery-based therapy. In the future, the identified themes could also be used to develop behavioral tasks focused on idiosyncratic and hence more emotionally relevant mental imagery content.

## Conclusions

In this paper, we provide an insight into PPMIs young people generated in their daily lives during the COVID-19 pandemic and the rates of pleasure intensity, vividness, ease of generation, and anticipation for each theme. We identified correlations between these PPMI characteristics and investigated associations between these characteristics and the diminishment of depressive symptoms. Our results suggest that vividness and ease of generation of PPMIs are associated with reduction of depressive symptoms, while anticipation and pleasure do not moderate the diminishment of depressive symptoms. As no reports on thematic analysis of PPMIs have been published previously, we believe that reporting and summarizing the most common PPMIs and their characteristics associated with the reduction of depressive symptoms, makes a unique contribution to mental imagery-related research. However, more studies are needed to provide a more in-depth overview of PPMIs and explore their characteristics, first, in experimental and natural settings with the general population and including data from different age groups, genders, and nationalities, and, afterwards, in therapeutic settings with clinical samples comprising patients with mental health symptoms.

## Electronic Supplementary Material

Below is the link to the electronic supplementary material.

Supplementary Material 1

## Data Availability

The datasets generated during and/or analyzed during the current study are not publicly available due to the decision of the ethical committee but are available from the corresponding author on reasonable request.

## References

[CR1] Anderson RJ, Evans GL (2015). Mental time travel in dysphoria: Differences in the content and subjective experience of past and future episodes. Consciousness and Cognition.

[CR2] Atance CM, O’Neill DK (2001). Episodic future thinking. Trends in Cognitive Sciences.

[CR3] Beck AT, Steer RA, Ball R, Ranieri W (1996). Comparison of Beck Depression inventories -IA and -II in psychiatric outpatients. Journal of Personality Assessment.

[CR4] Blackwell SE, Browning M, Mathews A, Pictet A, Welch J, Davies J, Watson P, Geddes JR, Holmes EA (2015). Positive imagery-based cognitive Bias Modification as a web-based Treatment Tool for depressed adults: A Randomized Controlled Trial. Clinical Psychological Science.

[CR5] Blouin-Hudon EMC, Pychyl TA (2017). A Mental Imagery intervention to increase future self-continuity and reduce procrastination. Applied Psychology.

[CR6] Bogdan R, Pizzagalli DA (2006). Acute stress reduces reward responsiveness: Implications for Depression. Biological Psychiatry.

[CR7] Boland J, Riggs KJ, Anderson RJ (2018). A brighter future: The effect of positive episodic simulation on future predictions in non-depressed, moderately dysphoric & highly dysphoric individuals. Behaviour Research and Therapy.

[CR8] Bourmistrova NW, Solomon T, Braude P, Strawbridge R, Carter B (2022). Long-term effects of COVID-19 on mental health: A systematic review. Journal of Affective Disorders.

[CR9] Braun V, Clarke V (2006). Using thematic analysis in psychology. Qualitative Research in Psychology.

[CR10] Chan CKY, Cameron LD (2012). Promoting physical activity with goal-oriented mental imagery: A randomized controlled trial. Journal of Behavioral Medicine.

[CR11] Diaz Hernandez L, Giezendanner S, Fischer R, Zeller A (2021). The effect of COVID-19 on mental well-being in Switzerland: A cross-sectional survey of the adult swiss general population. BMC Family Practice.

[CR12] Gobbi, S., Płomecka, M. B., Ashraf, Z., Radziński, P., Neckels, R., Lazzeri, S., Dedić, A., Bakalović, A., Hrustić, L., Skórko, B., Es haghi, S., Almazidou, K., Rodríguez-Pino, L., Alp, A. B., Jabeen, H., Waller, V., Shibli, D., Behnam, M. A., Arshad, A. H., & Jawaid, A. (2020). Worsening of Preexisting Psychiatric Conditions During the COVID-19 Pandemic. *Frontiers in Psychiatry*, *11*. 10.3389/fpsyt.2020.581426 10.3389/fpsyt.2020.581426 PMC7772353 33391049

[CR13] Grol M, Vanlessen N, De Raedt R (2017). Feeling happy when feeling down: The effectiveness of positive mental imagery in dysphoria. Journal of Behavior Therapy and Experimental Psychiatry.

[CR14] Hallford DJ, Barry TJ, Austin DW, Raes F, Takano K, Klein B (2020). Impairments in episodic future thinking for positive events and anticipatory pleasure in major depression. Journal of Affective Disorders.

[CR19] Holmes EA, Mathews A (2010). Mental imagery in emotion and emotional disorders. Clinical Psychology Review.

[CR18] Holmes EA, Lang TJ, Moulds ML, Steele AM (2008). Prospective and positive mental imagery deficits in dysphoria. Behaviour Research and Therapy.

[CR17] Holmes EA, Lang TJ, Deeprose C (2009). Mental Imagery and emotion in treatment across Disorders: Using the Example of Depression. Cognitive Behaviour Therapy.

[CR16] Holmes EA, Deeprose C, Fairburn CG, Wallace-Hadrill SMA, Bonsall MB, Geddes JR, Goodwin GM (2011). Mood stability versus mood instability in bipolar disorder: A possible role for emotional mental imagery. Behaviour Research and Therapy.

[CR15] Holmes EA, Blackwell SE, Burnett Heyes S, Renner F, Raes F (2016). Mental Imagery in Depression: Phenomenology, potential mechanisms, and treatment implications. Annual Review of Clinical Psychology.

[CR20] Ibrahim M (2012). Thematic analysis: A critical review of its process and evaluation. West East Journal of Social Sciences.

[CR22] Ji JL, Holmes EA, Blackwell SE (2017). Seeing light at the end of the tunnel: Positive prospective mental imagery and optimism in depression. Psychiatry Research.

[CR23] Ji JL, Kavanagh DJ, Holmes EA, MacLeod C, Di Simplicio M (2019). Mental imagery in psychiatry: Conceptual & clinical implications. CNS Spectrums.

[CR21] Ji JL, Geiles D, Saulsman LM (2021). Mental imagery-based episodic simulation amplifies motivation and behavioural engagement in planned reward activities. Behaviour Research and Therapy.

[CR24] Jorm AF, Christensen H, Henderson AS, Jacomb PA, Korten AE, Rodgers B (1998). Using the BIS/BAS scales to measure behavioural inhibition and behavioural activation: Factor structure, validity and norms in a large community sample. Personality and Individual Differences.

[CR25] Kalisch, R., Müller, M. B., & Tüscher, O. (2015). A conceptual framework for the neurobiological study of resilience. *Behavioral and Brain Sciences*, *38*. 10.1017/S0140525X1400082X 10.1017/S0140525X1400082X 25158686

[CR26] Karatzias T, Power K, Brown K, McGoldrick T (2009). Vividness of mental imagery in posttraumatic stress disorder (PTSD): The role of depression. Journal of Behavior Therapy and Experimental Psychiatry.

[CR27] Koo TK, Li MY (2016). A Guideline of selecting and reporting Intraclass correlation coefficients for Reliability Research. Journal of Chiropractic Medicine.

[CR28] Kosnes L, Whelan R, O’Donovan A, McHugh LA (2013). Implicit measurement of positive and negative future thinking as a predictor of depressive symptoms and hopelessness. Consciousness and Cognition.

[CR29] Kühner C, Bürger C, Keller F, Hautzinger M (2007). Reliabilität und Validität des revidierten Beck-Depressionsinventars (BDI-II): Befunde aus deutschsprachigen Stichproben. Der Nervenarzt.

[CR30] Lanata, A., Sebastiani, L., Di Gruttola, F., Di Modica, S., Scilingo, E. P., & Greco, A. (2020). Nonlinear Analysis of Eye-Tracking Information for Motor Imagery Assessments. *Frontiers in Neuroscience*, *13*. https://www.frontiersin.org/article/10.3389/fnins.2019.01431 10.3389/fnins.2019.01431 PMC6974582 32009892

[CR31] Landkroon E, van Dis EAM, Meyerbröker K, Salemink E, Hagenaars MA, Engelhard IM (2022). Future-oriented positive Mental Imagery reduces anxiety for exposure to Public speaking. Behavior Therapy.

[CR32] Linke J, Wessa M (2017). Mental Imagery Training increases wanting of rewards and reward sensitivity and reduces depressive symptoms. Behavior Therapy.

[CR33] MacLeod AK, Byrne A (1996). Anxiety, depression, and the anticipation of future positive and negative experiences. Journal of Abnormal Psychology.

[CR34] MacLeod AK, Tata P, Kentish J, Jacobsen H (1997). Retrospective and prospective cognitions in anxiety and depression. Cognition and Emotion.

[CR35] Marciniak, M. A., Shanahan, L., Myin-Germeys, I., Veer, I., Yuen, K., Binder, H., Walter, H., Hermans, E., Kalisch, R., & Kleim, B. (2022). *Imager – An mHealth mental imagery-based ecological momentary intervention targeting reward sensitivity: A randomized controlled trial*. PsyArXiv. 10.31234/osf.io/jn5u4 10.1111/aphw.12505 37942875

[CR36] Miles, M. B., & Huberman, A. M. (1994). *Qualitative data analysis: An expanded sourcebook, 2nd ed. - PsycNET*. APA PsycNET. https://psycnet.apa.org/record/1995-97407-000

[CR37] Morina N, Deeprose C, Pusowski C, Schmid M, Holmes EA (2011). Prospective mental imagery in patients with major depressive disorder or anxiety disorders. Journal of Anxiety Disorders.

[CR38] Morina N, Leibold E, Ehring T (2013). Vividness of general mental imagery is associated with the occurrence of intrusive memories. Journal of Behavior Therapy and Experimental Psychiatry.

[CR39] Nagels-Coune, L., Benitez-Andonegui, A., Reuter, N., Lührs, M., Goebel, R., De Weerd, P., Riecke, L., & Sorger, B. (2020). Brain-Based Binary Communication Using Spatiotemporal Features of fNIRS Responses. *Frontiers in Human Neuroscience*, *14*. 10.3389/fnhum.2020.00113 10.3389/fnhum.2020.00113 PMC7174771 32351371

[CR40] Paivio A (1969). Mental imagery in associative learning and memory. Psychological Review.

[CR41] Pictet A, Coughtrey AE, Mathews A, Holmes EA (2011). Fishing for happiness: The effects of generating positive imagery on mood and behaviour. Behaviour Research and Therapy.

[CR42] Pile V, Williamson G, Saunders A, Holmes EA, Lau JYF (2021). Harnessing emotional mental imagery to reduce anxiety and depression in young people: An integrative review of progress and promise. The Lancet Psychiatry.

[CR43] Pinheiro, J., Bates, D., DebRoy, S., Sarkar, D., Heisterkamp, S., Van Willigen, B., & Ranke, J. (2021). *nlme: Linear and Nonlinear Mixed Effects Models* (3.1–152). https://CRAN.R-project.org/package=nlme

[CR44] Renner F, Ji JL, Pictet A, Holmes EA, Blackwell SE (2017). Effects of engaging in repeated Mental Imagery of Future positive events on behavioural activation in individuals with major depressive disorder. Cognitive Therapy and Research.

[CR45] Renner, F., Werthmann, J., Paetsch, A., Bär, H. E., Heise, M., & Bruijniks, S. J. E. (2021). Prospective Mental Imagery in Depression: Impact on reward Processing and reward-motivated Behaviour. *Clinical Psychology in Europe*, *3*(2), 10.32872/cpe.3013. 10.32872/cpe.3013 PMC9667131 36397959

[CR46] Roepke AM, Seligman MEP (2016). Depression and prospection. British Journal of Clinical Psychology.

[CR47] Sampogna, G., Pompili, M., & Fiorillo, A. (2022). Mental Health in the time of COVID-19 pandemic: A Worldwide Perspective. *International Journal of Environmental Research and Public Health*, *19*(1), 10.3390/ijerph19010161. 10.3390/ijerph19010161 PMC8750501 35010419

[CR48] Skodzik T, Leopold A, Ehring T (2017). Effects of a training in mental imagery on worry: A proof-of-principle study. Journal of Anxiety Disorders.

[CR49] Stöber J (2000). Prospective cognitions in anxiety and depression: Replication and methodological extension. Cognition and Emotion.

[CR50] Strobel A, Beauducel A, Debener S, Brocke B (2001). Eine deutschsprachige Version des BIS/BAS-Fragebogens von Carver und White. [A german version of Carver and White’s BIS/BAS scales]. Zeitschrift Für Differentielle Und Diagnostische Psychologie.

[CR51] Szőllősi Á, Pajkossy P, Racsmány M (2015). Depressive symptoms are Associated with the phenomenal characteristics of imagined positive and negative future events. Applied Cognitive Psychology.

[CR52] Tiba, A. I. (2018). Feelings-As-Embodied Information: Studying the Role of Feelings As Images in Emotional Disorders. *Frontiers in Psychology*, *9*. https://www.frontiersin.org/article/10.3389/fpsyg.2018.00186 10.3389/fpsyg.2018.00186 PMC5826288 29515498

[CR53] Torkan H, Blackwell SE, Holmes EA, Kalantari M, Neshat-Doost HT, Maroufi M, Talebi H (2014). Positive imagery cognitive Bias modification in treatment-seeking patients with Major Depression in Iran: A pilot study. Cognitive Therapy and Research.

[CR54] Veer, I. M., Riepenhausen, A., Zerban, M., Wackerhagen, C., Puhlmann, L. M. C., Engen, H., Köber, G., Bögemann, S. A., Weermeijer, J., Uściłko, A., Mor, N., Marciniak, M. A., Askelund, A. D., Al-Kamel, A., Ayash, S., Barsuola, G., Bartkute-Norkuniene, V., Battaglia, S., Bobko, Y., & Kalisch, R. (2021). Psycho-social factors associated with mental resilience in the Corona lockdown. *Translational Psychiatry*, *11*(1), 10.1038/s41398-020-01150-4. 10.1038/s41398-020-01150-4 PMC7817958 33479211

[CR55] Watanabe, E., Fukuda, S., Hara, H., & Maeda, Y. (2006). Differences in relaxation by means of guided imagery in a healthy community sample.Alternative Therapies in Health and Medicine, *12*(2). 16541998

[CR56] Wilson AC, Schwannauer M, McLaughlin A, Ashworth F, Chan SWY (2018). Vividness of positive mental imagery predicts positive emotional response to visually presented Project Soothe pictures. British Journal of Psychology.

