# Supplementary material for: Positive Prospective Mental Imagery Characteristics in Young Adults and Their Associations with Depressive Symptoms
Source: Cognit Ther Res. 2023 Apr 28:1–12. Online ahead of print. doi: 10.1007/s10608-023-10378-5 (PMC10140715; doi:10.1007/s10608-023-10378-5)
Supplement: Supplementary file 1 — Supplementary Material 1 [file 10608_2023_10378_MOESM1_ESM.pdf]

Springer

the language of science

Conflict-of-Interest Disclosure Form

Cognitive Therapy and Research

When an author or the institution of the author has a relationship, financial or otherwise, with individuals or organizations that could influence the author's work inappropriately, a conflict of interest may exist. Examples of potential conflicts of interest may include but are not limited to academic, personal, or political relationships; employment; consultancies or honoraria; and financial connections, such as stock ownership and funding. Although an author may not feel that there are conflicts, disclosure of relationships and interests that could be viewed by others as conflicts of interest affords a more transparent and prudent process.

All authors and co-authors (if any) of papers submitted to *Cognitive Therapy and Research* must complete this form and disclose any actual or potential conflict of interest. The journal may publish such disclosures.

Please complete and return this form (one per author) and submit it/them **together with your manuscript** to the journal's Editorial Manager submission website.

[X] I have included a section, Conflict-of-Interest Statement, in the manuscript (applies even if there are no disclosures).

[X] I have no potential conflict of interest pertaining to this submission to *Cognitive Therapy and Research.*

Category for Disclosure	Description of Interest/Arrangement
	no conflict of interest.

Article Title Positive prospective mental imagery characteristics in young adults and their associations with depressive symptoms

All Authors Marta Anna Marciniak, Lilly Shanahan, Harald Binder, Raffael Kalisch, Birgit Kleim

Author Name BIRGIT KLEIM

Author Signature

Date 07/12/2022

Springer
http://www.springer.com/journal/10608

Cognitive Therapy and Research
Editor-in-Chief: Hofmann, S.G.
ISSN: 0147-5916 (print version)
ISSN: 1573-2819 (electronic version)
Journal no. 10608

Conflict-of-Interest Disclosure Form
Cognitive Therapy and Research

When an author or the institution of the author has a relationship, financial or otherwise, with individuals or organizations that could influence the author's work inappropriately, a conflict of interest may exist. Examples of potential conflicts of interest may include but are not limited to academic, personal, or political relationships; employment; consultancies or honoraria; and financial connections, such as stock ownership and funding. Although an author may not feel that there are conflicts, disclosure of relationships and interests that could be viewed by others as conflicts of interest affords a more transparent and prudent process.

All authors and co-authors (if any) of papers submitted to *Cognitive Therapy and Research* must complete this form and disclose any actual or potential conflict of interest. The journal may publish such disclosures.

Please complete and return this form (one per author) and submit it/them **together with your manuscript** to the journal's Editorial Manager submission website.

[X] I have included a section, Conflict-of-Interest Statement, in the manuscript (applies even if there are no disclosures).

[X] I have no potential conflict of interest pertaining to this submission to *Cognitive Therapy and Research.*

Category for Disclosure	Description of Interest/Arrangement

Article Title Positive prospective mental imagery characteristics in young adults and their associations with depressive symptoms

All Authors Marta Anna Marciniak, Lilly Shanahan, Harald Binder, Raffael Kalisch, Birgit Kleim

Author Name Marta Anna Marciniak

Author Signature Marta Marciniak Date 07/12/2022

Springer
http://www.springer.com/journal/10608

Cognitive Therapy and Research
Editor-in-Chief: Hofmann, S.G.
ISSN: 0147-5916 (print version)
ISSN: 1573-2819 (electronic version)
Journal no. 10608

Springer
the language of science

Conflict-of-Interest Disclosure Form
Cognitive Therapy and Research

When an author or the institution of the author has a relationship, financial or otherwise, with individuals or organizations that could influence the author's work inappropriately, a conflict of interest may exist. Examples of potential conflicts of interest may include but are not limited to academic, personal, or political relationships; employment; consultancies or honoraria; and financial connections, such as stock ownership and funding. Although an author may not feel that there are conflicts, disclosure of relationships and interests that could be viewed by others as conflicts of interest affords a more transparent and prudent process.
All authors and co-authors (if any) of papers submitted to *Cognitive Therapy and Research* must complete this form and disclose any actual or potential conflict of interest. The journal may publish such disclosures.
Please complete and return this form (one per author) and submit it/them **together with your manuscript** to the journal's Editorial Manager submission website.

☒ I have included a section, Conflict-of-Interest Statement, in the manuscript (applies even if there are no disclosures).

☐ I have no potential conflict of interest pertaining to this submission to *Cognitive Therapy and Research.*

**Category for Disclosure**	**Description of Interest/Arrangement**
honoraria/consultancy	RK has received advisory honoraria from JoyVentures, Herzlia, Israel

Article Title Positive prospective mental imagery characteristics in young adults and their associations with depressive symptoms

All Authors Marta Anna Marciniak, Lilly Shanahan, Harald Binder, Raffael Kalisch, Birgit Kleim

Author Name Raffael Kalisch

Author Signature [signature] Date 07/12/2022

Springer
http://www.springer.com/journal/10608

Cognitive Therapy and Research
Editor-in-Chief: Hofmann, S.G.
ISSN: 0147-5916 (print version)
ISSN: 1573-2819 (electronic version)
Journal no. 10608

Springer
the language of science

Conflict-of-Interest Disclosure Form

Cognitive Therapy and Research

When an author or the institution of the author has a relationship, financial or otherwise, with individuals or organizations that could influence the author's work inappropriately, a conflict of interest may exist. Examples of potential conflicts of interest may include but are not limited to academic, personal, or political relationships; employment; consultancies or honoraria; and financial connections, such as stock ownership and funding. Although an author may not feel that there are conflicts, disclosure of relationships and interests that could be viewed by others as conflicts of interest affords a more transparent and prudent process.

All authors and co-authors (if any) of papers submitted to *Cognitive Therapy and Research* must complete this form and disclose any actual or potential conflict of interest. The journal may publish such disclosures.

Please complete and return this form (one per author) and submit it/them **together with your manuscript** to the journal's Editorial Manager submission website.

[X] I have included a section, Conflict-of-Interest Statement, in the manuscript (applies even if there are no disclosures).

[X] I have no potential conflict of interest pertaining to this submission to *Cognitive Therapy and Research.*

Category for Disclosure	Description of Interest/Arrangement

Article Title Positive prospective mental imagery characteristics in young adults and their associations with depressive symptoms

All Authors Marta Anna Marciniak, Lilly Shanahan, Harald Binder, Raffael Kalisch, Birgit Kleim

Author Name Lilly Shanahan

Author Signature Lilly Shanahan Date 07/12/2022

http://www.springer.com/journal/10608

Cognitive Therapy and Research
Editor-in-Chief: Hofmann, S.G.
ISSN: 0147-5916 (print version)
ISSN: 1573-2819 (electronic version)
Journal no. 10608

Springer
the language of science

Conflict-of-Interest Disclosure Form
Cognitive Therapy and Research

When an author or the institution of the author has a relationship, financial or otherwise, with individuals or organizations that could influence the author's work inappropriately, a conflict of interest may exist. Examples of potential conflicts of interest may include but are not limited to academic, personal, or political relationships; employment; consultancies or honoraria; and financial connections, such as stock ownership and funding. Although an author may not feel that there are conflicts, disclosure of relationships and interests that could be viewed by others as conflicts of interest affords a more transparent and prudent process.

All authors and co-authors (if any) of papers submitted to *Cognitive Therapy and Research* must complete this form and disclose any actual or potential conflict of interest. The journal may publish such disclosures.

Please complete and return this form (one per author) and submit it/them **together with your manuscript** to the journal's Editorial Manager submission website.

[X] I have included a section, Conflict-of-Interest Statement, in the manuscript (applies even if there are no disclosures).

[X] I have no potential conflict of interest pertaining to this submission to *Cognitive Therapy and Research*.

Category for Disclosure	Description of Interest/Arrangement

Article Title Positive prospective mental imagery characteristics in young adults and their associations with depressive symptoms

All Authors Marta Anna Marciniak, Lilly Shanahan, Harald Binder, Raffael Kalisch, Birgit Kleim

Author Name Harald Binder

Author Signature [signature] Date 07/12/2022
